# Electrochemically Generated Acid and Its Containment to 100 Micron Reaction Areas for the Production of DNA Microarrays

**DOI:** 10.1371/journal.pone.0000034

**Published:** 2006-12-20

**Authors:** Karl Maurer, John Cooper, Marcelo Caraballo, James Crye, Dominic Suciu, Andrey Ghindilis, Joseph A. Leonetti, Wei Wang, Francis M. Rossi, Axel G. Stöver, Christopher Larson, Hetian Gao, Kilian Dill, Andy McShea

**Affiliations:** CombiMatrix Corporation, Mukilteo Washington, United States of America; Deutsches Krebsforschungszentrum, Germany

## Abstract

An addressable electrode array was used for the production of acid at sufficient concentration to allow deprotection of the dimethoxytrityl (DMT) protecting group from an overlaying substrate bound to a porous reaction layer. Containment of the generated acid to an active electrode of 100 micron diameter was achieved by the presence of an organic base. This procedure was then used for the production of a DNA array, in which synthesis was directed by the electrochemical removal of the DMT group during synthesis. The product array was found to have a detection sensitivity to as low as 0.5 pM DNA in a complex background sample.

## Introduction

**Figure 1:**
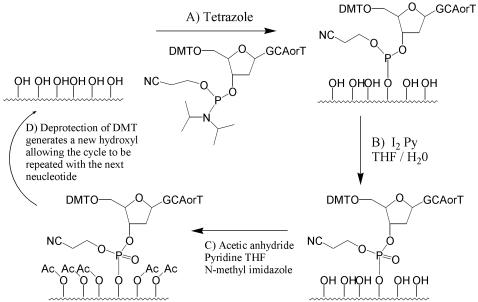
DNA Synthesis Cycle

Rapid developments in the field of DNA arrays have led to a number of methods for their *in situ* synthetic preparation, including photolithography using both fixed and programmable masks, ink jet printing of reagents, and electrochemical deprotection techniques.[1] These techniques have led to a number of commercially available DNA microarray products, including the CombiMatrix product used in this report as well as systems produced by Affymetrix, Agilent, Nimblegen, and Febit. These systems rely on the standard phosphoramidite DNA synthesis procedure, as shown in figure 1, with some modification of the deprotection or coupling procedures. The synthetic cycle for the production of DNA on CPG (controlled pore glass) columns is well known[2], [3] and allows rapid high fidelity synthesis of DNA oligonucleotides of whichever sequence is desired of up to 100 or more nucleotides in length. The challenge of constructing a DNA array, therefore, becomes how to contain the synthetic reactions to a very small (5–100 micron scale) area of space. This problem was first solved by Fodor in 1991[4] by the use of photolabile protecting groups and a masking strategy reminiscent of microchip production techniques. Unfortunately, this method, while commercially viable, has two major drawbacks. First, reagents having the necessary photoclevable protecting groups require separate syntheses and hence do not benefit from the availability of standard DNA synthesis reagents. Second, the masks needed for array production are expensive and time consuming to produce. A significant advance was made in 2001 by Gao[5] with the use of photochemically generated acid for the deblocking procedure. This procedure allowed the use of standard DNA synthesis reagents having the dimethoxytrityl 5′ protecting group. The use of a micro mechanical array of mirrors has also been reported to replace individual masks in the photo process and thereby reduce costs. However, the precise alignment and many steps of production in such systems have led us to consider the generation of acid by an electrochemical means for the deprotection of the DMT group during the array synthesis process.

## Results and Discussion

The electrode array used for this work was a CombiMatrix CustomArray.[6]–[8] This unit consists of a semiconductor silicon chip with an array of 1024 individually addressable platinum electrodes and a overlaying porous layer with available hydroxyl groups[9], which form the attachment sites for our synthetic work. The electrodes are 92 µm in diameter, reside in a 16×64 grid, and can be set to a specified voltage via connection to a PC with appropriate software, allowing different patterns of on and off electrodes to be used at each step in the construction of the array. The overlaying porous reaction layer (PRL) provides a solid support for attachment of our product DNA, similar to the CPG used in the column of a commercial DNA synthesizer, while maintaining close proximity to the electrode. This close proximity is necessary since the electrode array will be the source of the reagent (acid) used in the deblocking step of the DNA synthesis (step D, figure 1). In previous work, the deblocking reagent was light, and it was contained to a specific region of the chip by masking techniques. As stated earlier, this necessitates the production of a mask and precise alignment at each deblocking step. In contrast, in the electrochemical case reagent generated by the electrode is able to diffuse into the PRL but is prevented from excessive diffusion by the presence of a buffering agent in the surrounding solution. Near the electrode, the buffer is overwhelmed, allowing reaction of the ECG reagent with the PRL bound substrate; but further away, the concentration of the diffusing reagent is insufficient to overcome the concentration of the buffering agent, and no reaction occurs. Since the selection of which areas of the array to deprotect is done electronically, no mask is utilized and no alignment is necessary.

The production of acid from an electrochemical reaction has been known for some time[10], [11]. The development of a system for the electrochemical generation of acid using a hydroquinone/benzoquinone system for the removal of the DMT group during DNA synthesis was reported by Southern in 2005[12], [13]. While Southern showed that the generation of an EGA (electrochemically generated acid) was possible for a macro scale linear electrode, this procedure was never miniaturized to the scale and multiplicity useful for the production of a DNA microarray. In his technique, Southern relies on the diffusion of reagents from their electrode of origin across a solution filled gap to react on an opposing solid glass surface. On the small scale needed for the production of a microarray, diffusion of the acid generated at an electrode to neighboring electrodes becomes a serious problem. Montgomery [9] reported a solution to this problem using a buffered aqueous system, (demonstration in supplementary material) however the presence of considerable water in the deprotection step can be problematic due to the need of anhydrous conditions in the coupling step of the overall procedure.

Using the electrochemical arrays described above, we have recently reported the development of an electrochemical system using diphenylhydrazine for the removal of the t-BOC protecting group during peptide synthesis [14], [15] and on spatially contained, Pd-mediated transformations such as the Wacker oxidation, [16] Heck reaction [17], alcohol oxidation [18], and Coumarin formation [19].

**Figure 2:**
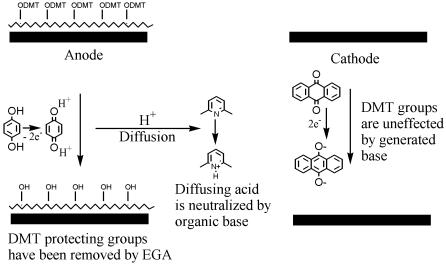
EGA Reation Scheme

As a non-aqueous alternative for acid generation, we have been studying the anodic oxidation of a hydroquinone/anthraquinone system (Figure 2). The oxidation of each mole of hydroquinone generates two moles of protons. To the mixture is added an organic base to limit the diffusion of protons to neighboring array elements. In this case, we chose a concentration of base below the concentration of acid that will be generated in the electrochemical reaction. In the area immediately surrounding an active electrode (anode), the amount of acid produced exceeds the amount of base present, and the generate acid is of sufficient strength to remove the DMT protecting group. As the acid diffuses away from this location, it encounters more base and, hence, is no longer able to remove the DMT group, thereby confining DNA synthesis to a discrete area on the array. Following the deprotection, standard DNA coupling strategies are employed to build upon the substrates that were selected for deprotection. With the containment of the synthesis achieved, the cycles are merely repeated, in each step deprotecting only where the addition of a new nucleotide is required, to generate an array of many different DNA oligonucleotides.

With the chemistry for deblocking in place, we next examined the synthesis of arrays using the generation of acid over different times and using different currents or voltages. It is expected that the use of higher currents or voltages will result in the generation of more acid and that the longer reaction time will provide more time for removal of the DMT group. However, the diffusion of reagents through the PRL of the microarray also occurs over time and can lead to loss of containment. For this reason, it would be useful if the deprotection step could be monitored. It is common practice during solid phase DNA synthesis to monitor DMT deprotection by quantitating the orange color of the DMT cation in the effluent methylene chloride/dichloroacetic acid deprotection solution. Unfortunately, due to the very small amount of material produced on an electrode in the microarray, as well as interfering absorptions in the electrochemical deprotection solution, we were not able to quantitate the removal of DMT directly. Instead we synthesized a known DNA oligomer over the electrodes and then relied on its hybridization with a complementary DNA strand bearing a fluorescent label. The label was then used to to visualize the quality of original synthesized DNA strand. The process of optimization of our deprotection conditions was iterative and is best exemplified by the formulation we have used in this report.

**Figure 3:**
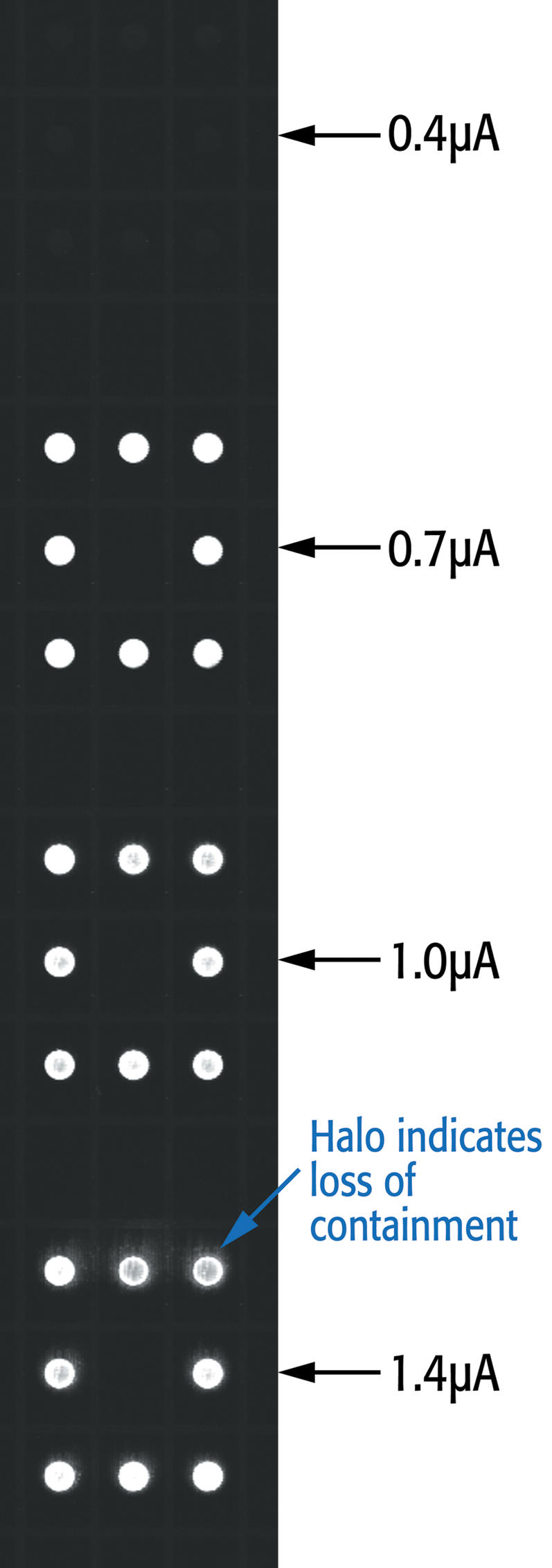
Example of a portion of an array with a box pattern of electrodes used for deprotection and synthesis at 0.4, 0.7, 1.0 and 1.4 micro amp/electrode.

We initially examined varying our deprotection current while holding the deprotection time constant at 1 minute. While no synthesis at all was observed at 0.4 microA per electrode (E), reasonable synthesis of a 20 mer probe occurred in a broad window from 0.7 to 1.4 microA /E. At the higher end of this current range, the acid is beginning to lose containment, as can be seen in Figure 3 by the halos surrounding the active electrodes. Insufficient deprotection could lead to the formation of deletions in a small proportion of the synthesized DNA, so the choice of the strongest deprotection conditions available is generally preferred. However, loss of containment, such as was seen at 1.4 microA/E, could lead to insertions at neighboring unused electrodes; hence, the highest current without halos was used for further work (1.0 microA/ electrode pair).

**Figure 4:**
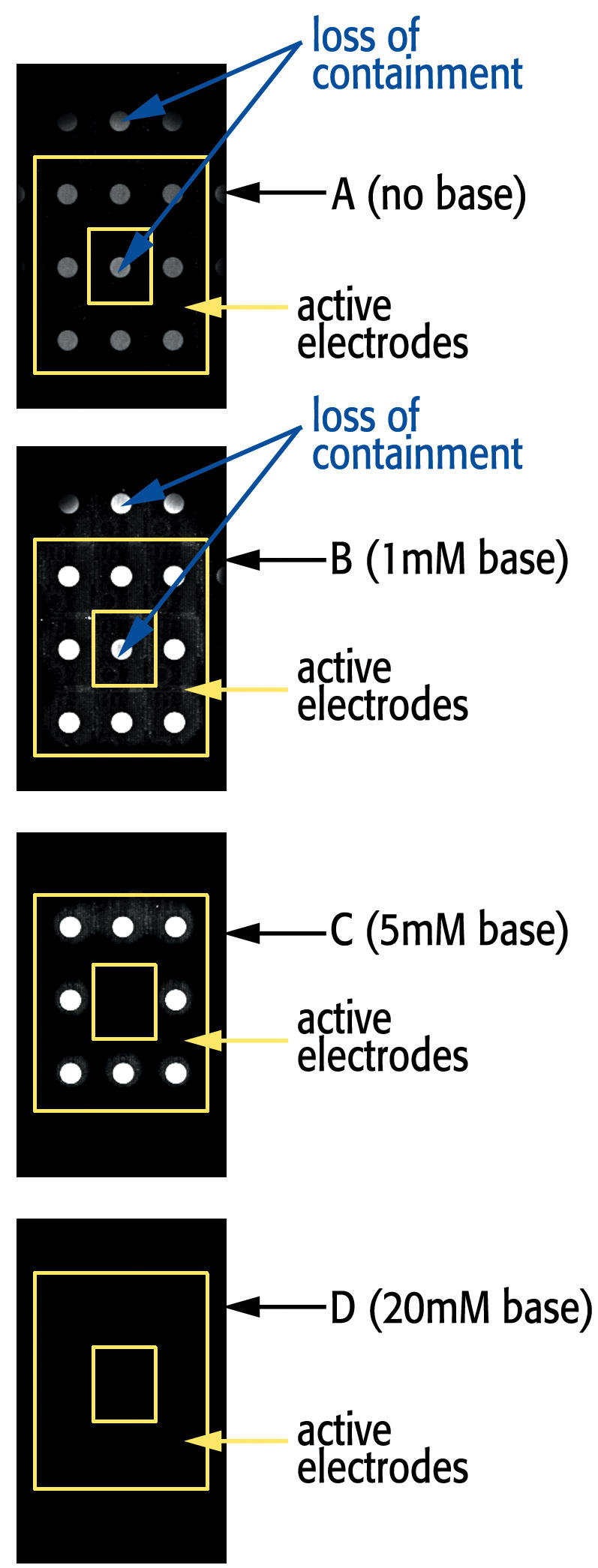
Example of a portion of an array with a box pattern of electrodes used for deprotection and synthesis with varying concentrations of base during the deprotection step. Note that the central electrode and electrodes surrounding the box are not active during deprotection and so should not have any synthesis occurring over them.

In a similar fashion, we optimized the time of deprotection by holding the current constant at 1 microA/ electrode and altering the time of the electrode is turned on to give a current tirtration. It was found that 1 minute gave an optimum spot shape and intensity while longer times led to degradation and shorter times to poor hybridization.

Finally, we examined variation in the concentration of base. On portions of a chip, oligomers were synthesized using electrochemical deblock with varying concentrations of 2,6-lutidine and our optimized conditions of 1 microA /E for 60 sec. The electrodes utilized for synthesis were arranged in box patterns with 8 peripheral electrodes on and the central and surrounding electrodes kept off. As can be seen in Figure 4, when no or low concentrations of base (1 µM) are used, the generated acid has sufficient concentration to diffuse beyond the desired electrodes, and synthesis occurs at the central electrode of the design and over the unused electrodes surrounding the box pattern (cases A and B). Use of 5 mM base appears to provide good containment, resulting in synthesis only at the 8 activated electrodes in the pattern (case C). When high concentrations (20 µM) of base are used, the entire deprotection reaction is stopped because the concentration of acid generated is unable to exceed the base present over the active electrodes (case D).

**Figure 5:**
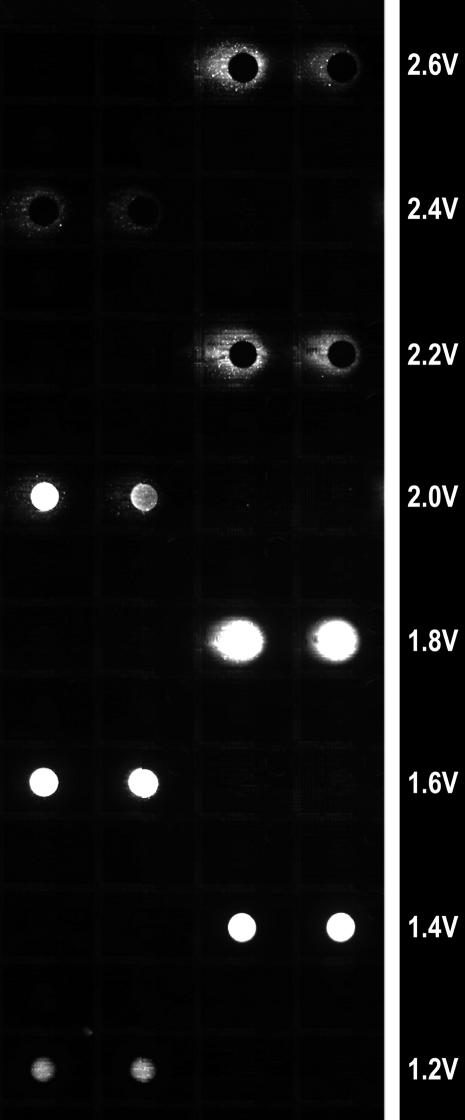
Example of a portion of an array demonstrating a voltage study from 1.2 to 2.4 V.

While synthesis is normally current sourced (current remains constant), it was also possible to electrochemically deblock using a voltage sourced system (voltage is held constant). The difference between these approaches was, again, investigated by running a series of synthetic reactions on the same chip; this time with varying voltages while base concentration was held constant at 5 mM and with 60 sec for reaction time. The voltage of this procedure was titrated from 1.2 V to 2.6 V in steps of 0.2 V, and the resulting DNA strand hybridized to its complement containing a fluorescent tag. As can be seen in Figure 5, lower voltages produced poor quality DNA, which was probably due to deletions resulting from incomplete deprotection. The optimum voltage centered at 1.8 V produced a well contained intense area of hybridization (representative of high quality DNA synthesis). Over 2.0 V, hybridization was, again, very poor which was, in this case probably due to damage to the overlaying porous support to which the synthesized DNA is immobilized.

**Figure 6:**
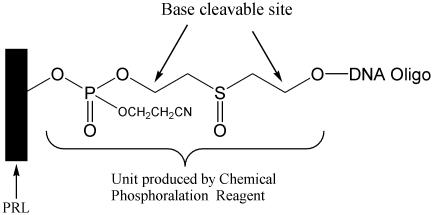
The design of the linker used in this experiment

**Figure 7:**
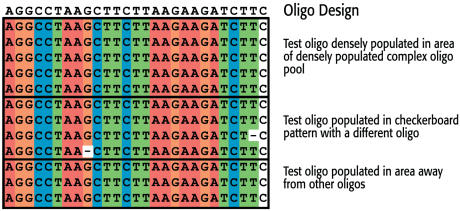
Results from the cloning and sequencing of DNA removed from the microarray.

With our synthesis conditions now optimized, we examined the quality of DNA produced on our array. To do this, we synthesized a DNA array with a cleavable linker on the 3′ end (closest to the chip) (Figure 6). This allowed us remove a sample of DNA for analysis. To determine if the fidelity of synthesis of a specific oligonucleotide is influenced by oligonucleotides produced with different sequences on neighboring electrodes, we designed areas on the chip where the designed oligomers were either surrounded by electrodes with different oligomers (complex), the neighboring electrodes were unused (checkerboard), or in an area away from other oligomers. The fidelity of our on-chip synthesized oligonucleotides was checked by Davis DNA sequencing. Briefly, the oligonucleotides were released from the semiconductor surface, amplified by PCR, cloned into a plasmid, colony-purified, and finally sequenced. A total of twelve sequencing reactions were performed. Two of the reactions showed single nucleotide deletions, whereas all other reactions were identical to the designed oligonucleotide sequence. Thus the average deletion rate was less than 1% in the samples tested (Figure 7).

**Figure 8:**
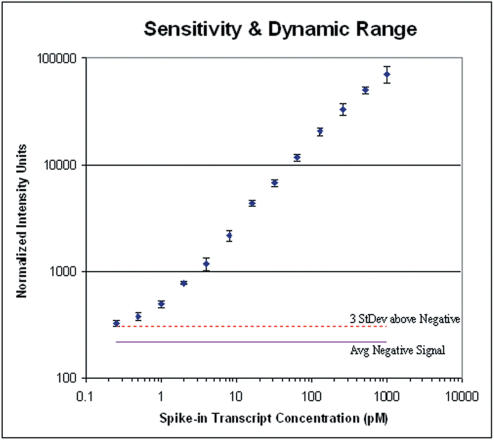
Hybridization analysis of 42 arrays demonstrating the sensitivity and linear dynamic range. Each point represents the mean of the normalized probe intensities for the spike-in control transcripts across three arrays plotted against the concentration of spike-in control transcripts. Error bars indicate the standard deviation across the three arrays at each data point. Spike-in control transcripts were added to a complex background of K-562 cRNA at 13 different concentrations ranging from 0.25 pM to 1024 pM (approximately 1∶600,000 to 1∶150 mass ratio) in two-fold increments. Sensitivity was determined to be signal detectable above the average signal for the same probes on a set of no-spike arrays (solid line) plus 3 standard deviations (dotted line).

Next, we produced a complex array of 35-mer length DNA oligos. To test the sensitivity of the array produced, the chip was tested by hybridization to a known fluorescent target in a complex cRNA background composed of cRNA from Human Leukemia cell (K-562 cell line). This complex background simulates the background seen during the standard use of a microarray. Varying concentrations of spike-in cRNA control transcripts were combined with a constant amount (150 nM) of K-562 cRNA complex background such that final concentration of spike-in control transcripts would range from 0.25 to 1024 pM in the hybridization solution. The samples were then fragmented and hybridized to the arrays, and the arrays were imaged to allow determination of the arrays detection limit. The arrays showed a 3 log dynamic range and sensitivity to as little at 1 part in 300,000 (0.5 pM) (Figure 8).

In conclusion, we have developed an electrochemical method for the removal of the DMT protecting group using electrochemically generated acid, which can be sufficiently confined so that it may be used for the construction of DNA oligonucleotides of different sequences on adjacent electrodes without the introduction of insertions from cross contaminating acid. The oligomers synthesized on the array were analyzed by cloning and sequencing and found to have no insertions and less than 1% deletions. Finally, when an array was produced using the electrochemical procedure and tested in an actual experimental setting, it was found to be of superior quality allowing observation of samples down to 0.5 pM in a complex background sample.

## Materials and Methods

### Procedures for array production

In general, the production of a DNA array is as follows: A CombiMatrix custom array chip was placed in the chamber of a DNA synthesizer programmed to run standard cyanoethyl phosphoramidite DNA synthesis. The acid deblocking reagent was replaced with an electrochemical deblocking mixture. The synthesis chamber allows the chip's electrode surface to be exposed to various solutions and allows electrical contact between the chip and a computer to determine which electrodes will be used at each stage of synthesis process. The counter electrode was a sheet of platinum on the opposing wall of the synthesis chamber. Once the synthesis was finished, the chip was removed from the chamber, deprotected chemically to remove terminal DMT protecting groups, and then exposed to ethylenediamine in ethanol (1∶1 v/v) for 1 hour at 65 deg C to remove side chain protective groups and cyanoethyl protecting groups.

### Preparation of electrochemical deblocking solution:

To a mixture of 400 mL methanol and 2.5 L acetonitrile was added hydroquinone (22 g), anthraquinone (2.0 g), tetraethylamonium p-toluene sulfonate (60 g). The mixture was stirred thoroughly, and 2,6-lutidine was added (2.32 mL), and the volume was brought to a total of 4.0 L with added acetonitrile and stirred until fully dissolved.

### Procedures for optimization studies study

A CombiMatrix custom array chip was synthesized using a pattern, which provided 4 to 8 separate areas of the chip to be used in a sequential manner. This allowed the machine to be set to a specified study parameter (current, voltage, or time) for the synthesis of each section of the chip while other sections were unaltered. After the DNA syntheses were complete for all sections of the chip (4 to 8 total runs), the chip was deprotected as above. A direct comparison of the resulting synthesized DNA was then possible following hybridization to the fluorescently labeled (Cy-5) complement.

### Procedures for cloning and sequencing analysis

For PCR, an array was synthesized using phosphate-on for the first cycle of synthesis (Glen research #10-1900-90), with two conserved priming sites flanking a variable region. Following synthesis, we exposed the semiconductor surface to 28.5% ammonium hydroxide for 4 h at 65°C to release synthesized oligonucleotides from the surface of the chip. After evaporating the ammonia in a speed-vac for one hour at 75°C, the dried pellet was dissolved in 25 µL nuclease-free distilled water, and desalted by passing through a G25 column (Amersham Biosciences), following the manufacturer's instructions. The oligonucleotides were amplified in 50 µL mixtures using two primers. One primer was an oligonucleotide resembling T7 RNA polymerase promoter 5′-TAATACGACTCACTATAGGG-3′, the other primer was complementary to a variable flanking sequence. Thirty cycles of PCR were performed as follows: 95°C for 30 s, 56°C for 30 s, 72°C for 45 s and one final cycle at 72°C for 7 min. Double-strand DNA (dsDNA) oligonucleotides, amplified by PCR as described above, were cloned into the pCR2.1-TOPO*TA vector (Invitrogen) and *E. coli* DH5α transformed and colony purified. Plasmid DNA was isolated and purified using Qiagen plasmid purification kit (Qiagen) and sequenced at Davis Sequencing (Davis, CA). The resulting sequences were aligned with Clone Manager (Scientific & Educational Software).

### Procedures for testing of a DNA microarray produced using this technique

Specific 35mer probes were created for various genes involved in immune system pathways, as well as a number of housekeeping genes. In addition, multiple probes were designed against long PCR fragments generated from phage Lambda. The microarray was designed with four replicates of each probe distributed across the array. The custom oligonucleotide arrays were synthesized using phosphoramidite chemistry under electrochemical control as detailed above. Forty-two arrays were assessed in this experiment (Arrays with obvious defects were removed from analysis).

Complex background sample was prepared from Human Leukemia, Chronic Myelogenous (K-562 cell line) poly A+ RNA (BD Biosciences, Palo Alto, CA) by a modified Eberwine amplification technique producing biotin-incorporated cRNA. Briefly, double-stranded cDNA, incorporating a T7 RNA Polymerase promoter site, was prepared from K-562 poly A+ RNA using the Roche Applied Science (Indianapolis, IN) Microarray cDNA Synthesis Kit. Double stranded cDNA was purified using a Qiagen (Valencia, CA) QIAquick purification kit. Biotin-labeled antisense RNA (cRNA) was produced using an Ambion (Austin, TX) MegaScript™ kit by combining biotin-UTP (Roche Applied Science, Indianapolis, IN) with unlabeled UTP in a molar ratio of 1∶3. The biotin-labeled cRNA was purified using a Qiagen RNeasy kit. Spectrophotometry measurements were used to calculate concentration and purity of the cRNA sample. In addition, integrity of the RNA was checked on a 2% agarose gel. Spike-in control transcripts were prepared using PCR fragments containing a T7 RNA Polymerase promoter site, as template for transcription.

Varying concentrations of spike-in cRNA control transcripts were combined with a constant amount (150 nM) of K-562 cRNA complex background such that final concentration of spike-in control transcripts would range from 0.25 to 1024 pM in the hybridization. The cRNA mixtures were fragmented in 1× fragmentation solution (40 mM Tris-Acetate, pH8.1, 100 mM KOAc, 30 mM MgOAc) at 95C for 20 minutes, then placed on ice. The microarrays were assembled with hybridization caps and rehydrated with RNase-free water at 65C for 10 minutes. After rehydrating, blocking solution (6× SSPE, 20 mM EDTA, 0.05% Tween-20, 5× Denhardt's Solution, 0.05% SDS, 100 ng/µL Sonicated Salmon Sperm DNA) was added, and the arrays were incubated at hybridization temperature (45C) for 30 minutes. The fragmented cRNA sample was added to hybridization solution (6× SSPE, 0.05% Tween-20, 20 mM EDTA, 25% DI Formamide, 0.05% SDS, 100 ng/µL Sonicated Salmon Sperm DNA) and denatured 3 minutes at 95C. Samples were placed briefly on ice followed by centrifugation at 13,000 × g for 3 min. Blocking solution was removed from the hybridization chamber, and 145 µL of hybridization solution was applied to the arrays. Hybridization was carried out in a Fisher Scientific Isotemp hybridization incubator for 18 h at 45°C under gentle rotation.

Following hybridization, arrays were washed at hybridization temperature for 5 minutes with 6× SSPE, 0.05% Tween-20 pre-warmed to 45°C. Washings continued with 3× SSPE, 0.05% Tween-20 for 5 minutes at room temperature (RT), 0.5× SSPE, 0.05% Tween-20 for 5 min at RT and 2× PBS, 0.1% Tween-20. Arrays were prepared for detection by blocking in 2× PBS, 0.1% Tween-20, 1% Acetylated BSA for 15 min at RT. Streptavidin-Alexa Fluor 647 (Molecular Probes, Eugene, OR) was diluted in blocking solution to a final concentration of 1 µg/mL, and arrays were incubated with this solution for 30 min at RT while protected from light. Final washing steps were performed at RT for 5 min each in 2× PBS, 0.1% Tween-20 followed by two rounds of 2× PBS with no detergent.

The 42 microarrays were imaged with Cy5 filter sets on an Applied Precision (Issaquah, WA) arrayWoRx™ Biochip Reader. Imaging was performed while the array was wet with 2× PBS under a LifterSlip™ glass cover slip (Erie Scientific, Portsmouth, NH). Images were analyzed using CombiMatrix web-based imaging software.

## Supporting Information

Example of acid containment in Aq system (0.02 MB DOC)

Acid containment video (3.26 MB SWF)
