# Supplementary material for: Electrochemically Generated Acid and Its Containment to 100 Micron Reaction Areas for the Production of DNA Microarrays
Source: PLoS One. 2006 Dec 20;1(1):e34. doi: 10.1371/journal.pone.0000034 (PMC1762329; doi:10.1371/journal.pone.0000034)
Supplement: Example of acid containment in Aq system — (0.02 MB DOC) [file pone.0000034.s001.doc]

As a demonstration of the ability of EGA to be contained to an area proximal to an active electrode on an array, an array coated with agarose as a PRL was saturated with a solution of 1M phosphate buffer at a pH of approximately 7 containing bromphenyl blue indicator dye. Currant was applied to the array in a checkerboard fashion of alternating cathodes and anodes and the results recorded through an optical microscope. A comparable experiment was than run lacking the buffering phosphate. As can be seen in the accompanying video the generation of acid is contained within the circular feature of the electrode (yellow color, vs. blue/purple bulk solution) while in the unbuffered case the acid diffuses significantly beyond its generation point.

Similar procedures have been used in organic systems, however the volatility of the organic solvents used precluded video documentation.
